# IL-11 prevents IFN-γ-induced hepatocyte death through selective downregulation of IFN-γ/STAT1 signaling and ROS scavenging

**DOI:** 10.1371/journal.pone.0211123

**Published:** 2019-02-19

**Authors:** Akimitsu Miyawaki, Yoshiko Iizuka, Hitomi Sugino, Yoshifumi Watanabe

**Affiliations:** Department of Pharmaceutical Sciences, Musashino University, Tokyo, Japan; Karolinska Institutet, SWEDEN

## Abstract

**Aims:**

Interferon-γ (IFN-γ) exhibits hepatotoxicity through signal transducer and activator of transcription 1 (STAT1) activation. On the contrary, interleukin-11 (IL-11) shows tissue-protective effects on various organs including the liver through STAT3 activation. Here, we found that IL-11 pretreatment protects hepatocytes from IFN-γ-induced death and investigated the molecular mechanisms, particularly focusing on signal crosstalk.

**Methods and results:**

Primary culture mouse hepatocytes were treated with IL-11 prior to IFN-γ, and cell death was evaluated by lactate dehydrogenase release into media. As a result, IL-11 pretreatment effectively suppressed IFN-γ-induced hepatocyte death. Since IFN-γ-induced hepatocyte death requires STAT1 signaling, the activity of STAT1 was analyzed. IFN-γ robustly activated STAT1 with its peak at 1 hr after stimulation, which was significantly attenuated by IL-11 pretreatment. Consistently, IL-11 pretreatment impeded mRNA increase of STAT1-downstream molecules promoting cell death, i.e., IRF-1, caspase 1, bak, and bax. IL-11-mediated suppression of STAT1 signaling was presumably due to upregulation of the suppressor of cytokine signaling (SOCS) genes, which are well-known negative feedback regulators of the JAK/STAT pathway. Interestingly, however, IFN-γ pretreatment failed to affect the following IL-11-induced STAT3 activation, although IFN-γ also upregulated SOCSs. Finally, we demonstrated that IL-11 pretreatment mitigated oxidative stress through increasing expression of ROS scavengers.

**Conclusion:**

IL-11 protects hepatocytes from IFN-γ-induced death via STAT1 signal suppression and ROS scavenging. Further investigation into the mechanisms underlying selective negative feedback regulation of IFN-γ/STAT1 signaling compared to IL-11/STAT3 signaling may shed new light on the molecular biology of hepatocytes.

## Introduction

The liver possesses a strong ability to regenerate itself after injury, compared to other organs. For example, 70% hepatectomy results in almost complete recovery in liver mass by 21 days post-operation in mice [1]. In contrast, however, the regenerative capacity of the liver is gradually exhausted in situations of cumulative damage, such as chronic virus infection and alcoholic/nonalcoholic steatohepatitis [2]. These pathologies lead to fibrosis and, eventually, cirrhosis/carcinogenesis of the liver, which is hardly reversible and requires liver transplantation [3]. Therefore, it is of great importance to protect liver parenchymal cells, namely hepatocytes, from chronic damage in order to prevent liver disease progression.

It is widely accepted that dysregulated inflammatory cytokine expression plays a pivotal role in the progression of chronic liver diseases [4]. Among the inflammatory cytokines, we have previously reported that interferon-gamma (IFN-γ) by itself exhibits hepatotoxic effects through upregulation of interferon regulatory factor-1 (IRF-1), a downstream proapoptotic molecule of IFN-γ/signal transducer and activator of transcription 1 (STAT1) signaling [5]. IFN-γ was originally identified as an antiviral agent and has since been found to possess pleiotropic immunomodulatory functions [6–8]. Recently, it has been reported that IFN-γ is upregulated in steatohepatitis without infection, contributing to augmentation of inflammatory responses and progression of the disease [9]. Therefore, protecting hepatocytes from IFN-γ-induced death has potential therapeutic implications in liver diseases.

Interleukin-11 (IL-11) is an IL-6 family cytokine but can exhibit anti-inflammatory properties unlike IL-6 [10,11]. Activating STAT3 upon binding to its receptor, IL-11 protects a variety of organs including the liver by suppressing inflammation. For example, IL-11 administration significantly attenuates acetaminophen-induced hepatic injury through downregulation of tumor necrosis factor-α (TNF-α) [12]. It has also been reported that IL-11 mitigates liver ischemia/reperfusion injury with decreased expression of proinflammatory cytokines [13,14]. In addition to its anti-inflammatory functions, IL-11/STAT3 signaling renders resistance against oxidative stress by upregulating reactive oxygen species (ROS) scavengers, such as manganese superoxide dismutase (MnSOD) and metallothioneins (MTs) [15,16]. In fact, IL-11 contributes to the reduction of oxidative stress in the acetaminophen-induced liver injury model [17].

Although the hepatoprotective roles of IL-11 have been recognized, its potential in restraining cytokine-induced hepatotoxicity remains unexplored. Hence, in this study, we investigated the effects of IL-11 on IFN-γ-induced hepatocyte death and found that IL-11-pretreated hepatocytes were resistant to the following IFN-γ stimulation. Since both cytokines activate the common Janus kinase (JAK)/STAT cascade, the mechanism of IL-11-mediated protection from IFN-γ was studied with an intensive focus on signal crosstalk in addition to ROS scavenging. The data we present here reveal new aspects of signal regulation in hepatocytes.

## Materials and methods

### Study approval

Animal care was conducted under the supervision of the Institutional Animal Experiments Committee of Musashino University. All experimental procedures conformed to the *Guide for the Care and Use of Laboratory Animals*: *Eighth Edition*, updated by the US National Research Council Committee in 2011, and were approved by the Institutional Animal Experiments Committee of Musashino University.

### Hepatocyte primary culture

Eight-ten-week old female ICR mice were euthanized by cervical dislocation under isoflurane anesthesia. Primary hepatocytes were obtained as described previously [18] with minor modifications. In brief, hepatic cells were isolated from the liver by perfusing 0.0125% collagenase type X (Wako, Osaka, JP), followed by passing through a 70-μm opening mesh. Hepatocytes were purified two times by centrifugation at 50 g for 2 min. Nonparenchymal cells in the supernatant were discarded. Dead hepatocytes were removed by density gradient centrifugation in 45% Percoll (GE Healthcare, Buckingham, GB) at 50 g for 10 min. The obtained hepatocytes were then suspended in William’s Medium E (Sigma, St. Louis, US) supplemented with 10% fetal bovine serum (FBS) (Japan Bio Serum, Fukuyama, JP) and seeded on collagen-coated dishes at 10^5^ cells/mL. The media were changed to William’s Medium E containing 2% FBS 3 hr after seeding.

### Evaluation of hepatocyte death

Hepatocyte death was evaluated by lactate dehydrogenase (LDH) release into media, which was measured with CytoTox 96 Non-Radioactive Cytotoxicity Assay (Promega, Madison, US) in accordance with the manufacturer’s protocol. Absorbance at 490 nm was measured by iMark Microplate Absorbance Reader (Bio-Rad, Hercules, US). For a positive control, hepatocytes were lysed with the attached Lysis Solution, and the activity of released LDH was considered to represent 100% cell death.

### Quantitative RT-PCR

Total RNA was obtained using QIAzol lysis reagent (QIAGEN, Hilden, DE), and complementary DNA (cDNA) was synthesized from the RNA using ReverTra Ace -α- (TOYOBO, Osaka, JP). The resulting cDNA was applied for following quantitative RT-PCR (qRT-PCR) experiments with THUNDERBIRD SYBR qPCR Mix (TOYOBO, Osaka, JP) and the 7500 Fast Real-Time PCR System (Applied Biosystems, Foster City, US). All the procedures were conducted in accordance with the manufacturer’s protocol. The primer pairs used are listed below.

β-actin

forward: 5′-CATCCGTAAAGACCTCTATGCCAAC-3′,

reverse: 5′-ATGGAGCCACCGATCCACA-3′.

bak

forward: 5′-CAACCCCGAGATGGACAACTT-3′,

reverse: 5′-CGTAGCGCCGGTTAATATCAT-3′.

bax

forward: 5′-TGAAGACAGGGGCCTTTTTG-3′,

reverse: 5′-AATTCGCCGGAGACACTCG-3′.

bim

forward: 5′-CCCGGAGATACGGATTGCAC-3′,

reverse: 5′-GCCTCGCGGTAATCATTTGC-3′.

caspase 1

forward: 5′-ACAAGGCACGGGACCTATG-3′,

reverse: 5′-TCCCAGTCAGTCCTGGAAATG-3′.

IRF-1

forward: 5′-TCACACAGGCCGATACAAAG-3′,

reverse: 5′-CACAACGGAAGTTTGCCT-3′.

MnSOD [19]

forward: 5′-CTTCAATAAGGAGCAAGGTCG-3′,

reverse: 5′-TGAAGGTAGTAAGCGTGCTC-3′.

MT1 [19]

forward: 5′-CGTAGCTCCAGCTTCACCAGATCTC-3′,

reverse: 5′-TGGTGGCAGCGCTGTTCGT-3′.

MT2 [19]

forward: 5′-GCTTTTGCGCTCGACCCAATACTCTC-3′,

reverse: 5′-GGAGCAGCAGCTTTTCTTGCAGGAAG-3′.

SOCS1

forward: 5′-CTGCGGCTTCTATTGGGGAC-3′,

reverse: 5′-AAAAGGCAGTCGAAGGTCTCG-3′.

SOCS3

forward: 5′-ATGGTCACCCACAGCAAGTTT-3′,

reverse: 5′-TCCAGTAGAATCCGCTCTCCT-3′.

### Immunoblot analysis

Hepatocytes were scraped in buffer containing 150 mmol/L NaCl, 50 mmol/L Tris-HCl (pH 6.8), 1% Triton X-100, 0.1% SDS, 1 mM dithiothreitol, phosphatase inhibitor cocktail (Nacalai Tesque, Kyoto, JP), and protease inhibitor cocktail (Wako, Osaka, JP). After denaturing at 96°C for 6 min, the resulting protein samples were separated by SDS-PAGE and transferred onto a polyvinylidene difluoride membrane (Millipore, Burlington, US). Immunoblotting was performed with the following primary antibodies at 1/500 or 1/1000 (for β-actin only). β-actin: PM053-7 (MBL, Nagoya, JP). STAT1: 14994 (CST, Danvers, US). pSTAT1: 7649 (CST, Danvers, US). STAT3: 4904 (CST, Danvers, US). pSTAT3: 9145 (CST, Danvers, US). SOCS1: sc-518028 (Santa Cruz Biotechnology, Dallas, US). SOCS3: ab16030 (Abcam, Cambridge, UK). HRP-conjugated goat anti-rabbit IgG (CST, Danvers, US) was applied at 1/1000 as secondary antibody. Chemiluminescence of Clarity Western ECL Substrate (Bio-Rad, Hercules, US) or ECL Prime Western Blotting Detection Reagent (GE Healthcare, Buckingham, GB) was detected by ImageQuant LAS 4000 (GE Healthcare, Buckingham, GB).

### Cytokine stimulation

Hepatocytes were stimulated by IFN-γ (PeproTech, Rocky Hill, US) or IL-11 (PeproTech, Rocky Hill, US) at 100 U/mL or 100 ng/mL, respectively. For JAK inhibition, 2.5 μM Ruxolitinib (SYNkinase, Parkville, AU) was applied.

### Dihydroethidium assay

Hepatocytes were pretreated with 100 ng/mL IL-11. Sixteen hr after IL-11 pretreatment, the cells were stimulated by 100 U/mL IFN-γ. Dihydroethidium (DHE) (Cayman, Ann Arbor, US) at 20 μM was added to the media 8 hr after the IFN-γ stimulation and incubated for 30 min, followed by substituting the media to PBS and microscopic observation with BZ-X710 (Keyence, Osaka, JP). Pictures were taken from the center of wells of a 96-well culture plate with a 4x objective lens. The capture area of each picture was approximately 0.13 cm^2^. Since hepatocytes were seeded at 10000 cells/well and bottom area of each well was 0.32 cm^2^, approximately 4000 cells were estimated to be within a picture. Fluorescence intensity of four independent pictures (approximately 16000 cells in total) was calculated for each experimental group. In the ImageJ analysis, the entire area of each picture was surrounded with a square and fluorescence within the square was quantified.

### Statistics

For comparison between two groups, Welch’s *t* test was applied. Multiple groups were compared with one-way ANOVA, followed by post-hoc tests. The statistical significance level was set at *P* < 0.05. Data were presented as mean ± S.D. and specified in S1 Table.

## Results

### IFN-γ-induced hepatocyte death was mediated by JAK/STAT1 signaling

**Fig 1:**
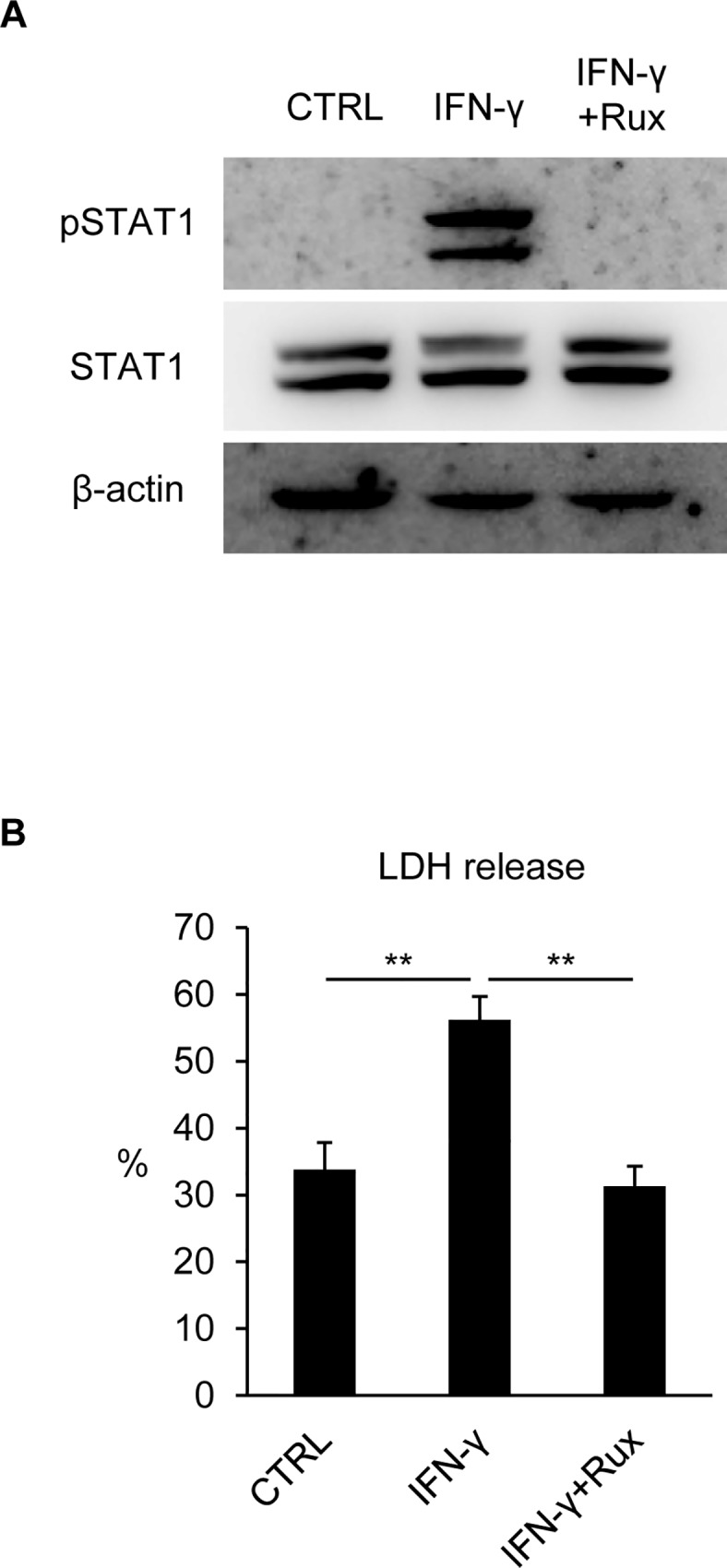
JAK/STAT1 signaling was required for IFN-γ-induced hepatocyte death. Hepatocytes were stimulated by IFN-γ with/without Ruxolitinib (Rux). (A) Protein samples were collected 1 hr after stimulation and subjected to immunoblotting with anti-β-actin, STAT1, and pSTAT1 antibodies. (B) LDH release was analyzed 48 hr after stimulation. LDH release from Lysis Solution (supplied in kit)-treated cells was referred to as 100% cell death. Data were shown as mean ± S.D. n = 5. One-way ANOVA, **P < 0.01.

We have previously shown that IFN-γ induces hepatocyte death via induction of interferon regulatory factor-1 (IRF-1), which is an apoptosis-promoting transcription factor upregulated by STAT1 [5]. In order to confirm that the JAK/STAT1 pathway is responsible for the IFN-γ-induced hepatocyte death, hepatocytes were treated with IFN-γ in combination with Ruxolitinib (Rux), a JAK1/JAK2 inhibitor. As a result, Rux completely blocked STAT1 activation (Fig 1A) and an increase in LDH release from hepatocytes (Fig 1B) induced by IFN-γ, verifying that STAT1 activation is indispensable for hepatocyte death.

### IL-11 pretreatment prevented IFN-γ-induced hepatocyte death

**Fig 2:**
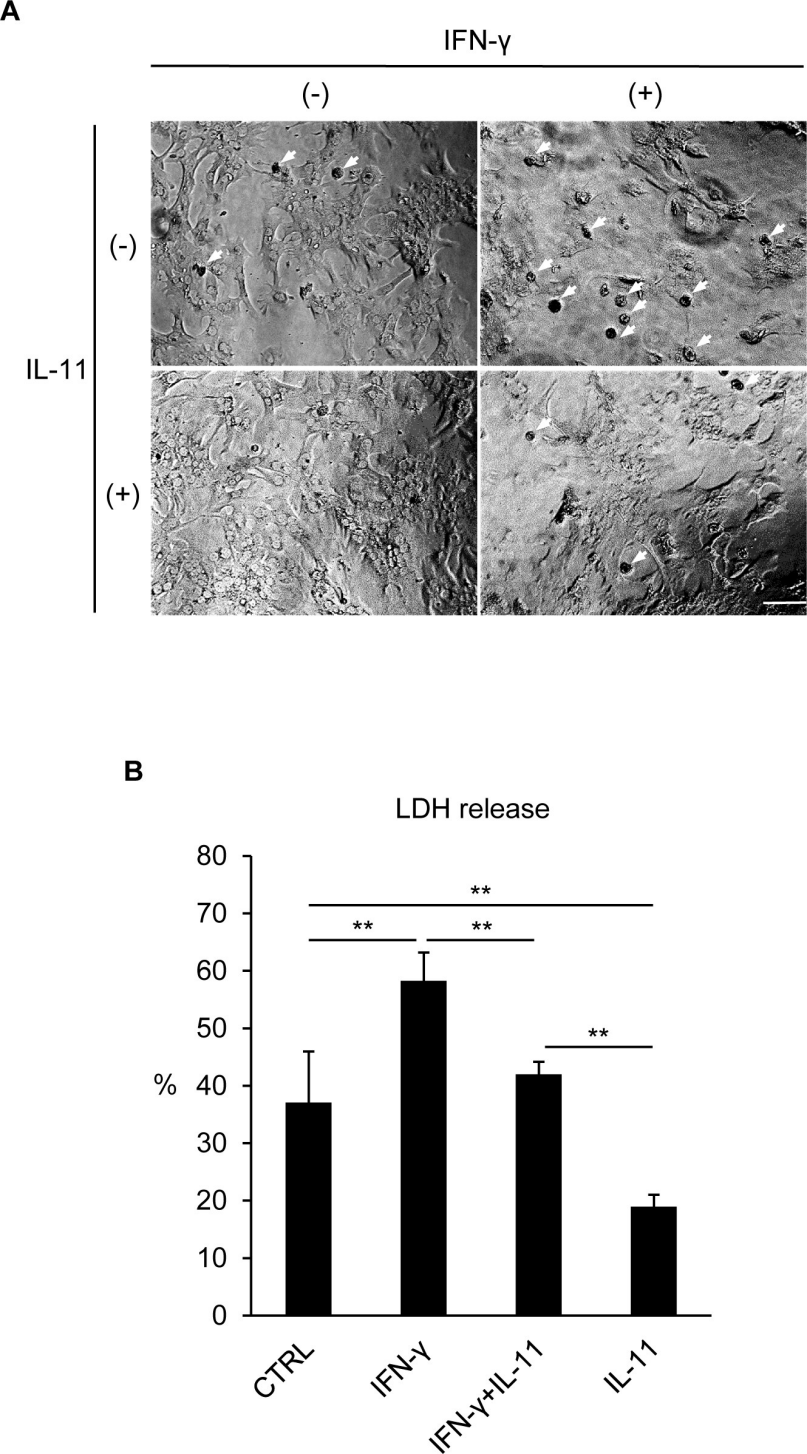
IFN-γ-induced hepatocyte death was mitigated by IL-11 pretreatment. Hepatocytes were pretreated with IL-11, followed by IFN-γ stimulation 16 hr after IL-11 pretreatment. (A) Microscopic observation was performed 48 hr after IFN-γ stimulation. Bar: 100 μm. Arrows: apoptotic bodies. (B) LDH release was analyzed 48 hr after IFN-γ stimulation. Data were shown as mean ± S.D. n = 4. One-way ANOVA, **P < 0.01.

Since IL-11 exerts many of its functions via induction of transcriptional activation by STAT3 [20], we pretreated hepatocytes with IL-11 prior to IFN-γ stimulation to evaluate its effects. Hepatocytes, when stimulated with IFN-γ, formed apoptotic bodies, which were apparently suppressed by IL-11 pretreatment (Fig 2A). Consistently, LDH release from hepatocytes was significantly reduced and Caspase 3 cleavage became slightly milder in the IL-11-pretreated groups (Fig 2B; S1 Fig). It may be of note that IL-11 not only inhibited IFN-γ-induced death but also hampered the baseline death of hepatocytes, which is in line with previous reports describing the broad spectrum of cytoprotective functions of STAT3 [21,22].

### IL-11 pretreatment suppressed STAT1 signal activation by IFN-γ

**Fig 3:**
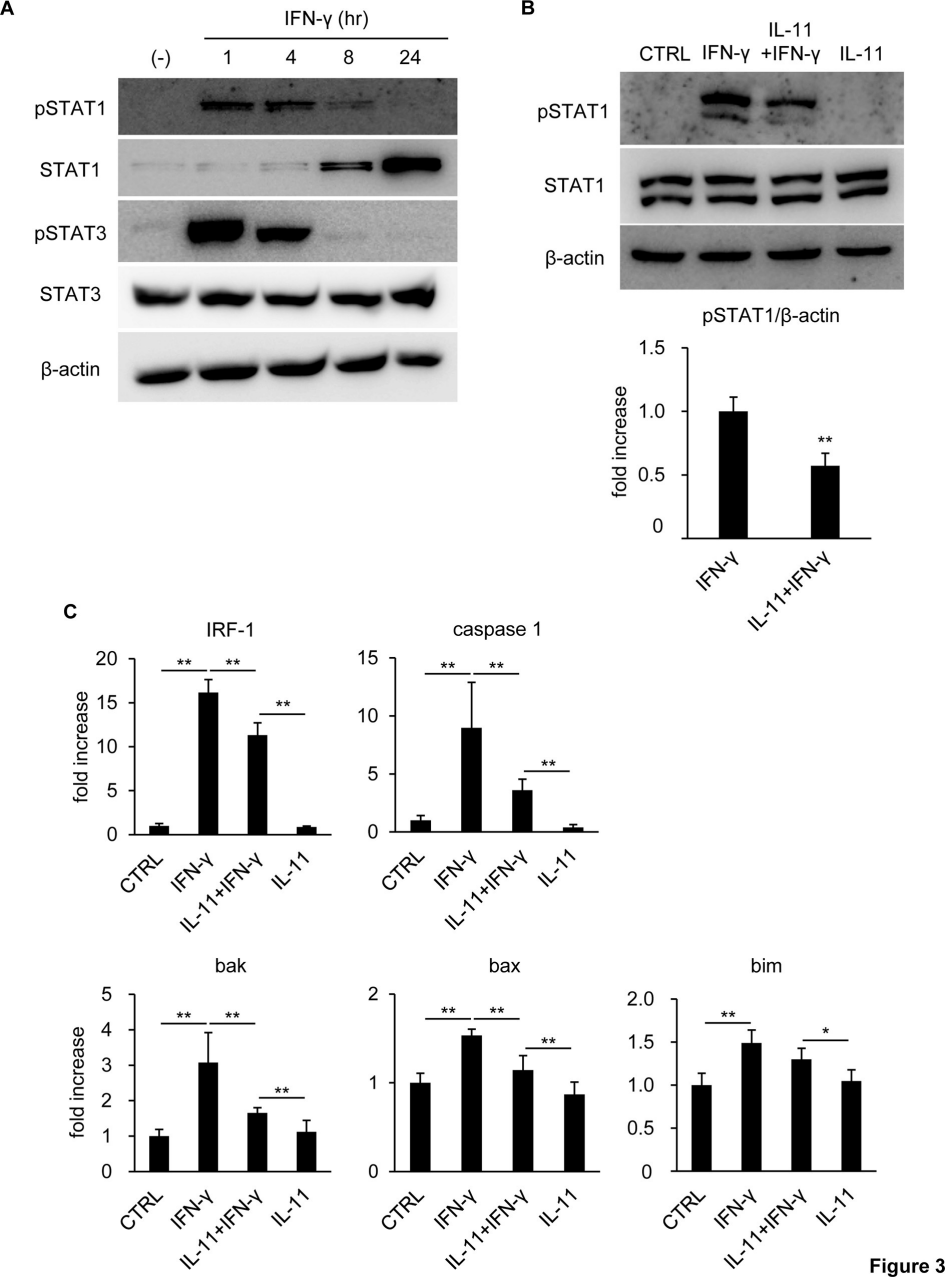
IFN-γ-induced STAT1 activation was suppressed by IL-11 pretreatment. (A) Hepatocytes were stimulated by IFN-γ. Protein samples were collected at the indicated time points after stimulation and subjected to immunoblotting with anti-β-actin, STAT1, pSTAT1, STAT3, and pSTAT3 antibodies. (B, C) Hepatocytes were pretreated with IL-11, followed by IFN-γ stimulation 16 hr after IL-11 pretreatment. (B) Protein samples collected 1 hr after IFN-γ stimulation were subjected to immunoblotting with anti-β-actin, STAT1, and pSTAT1 antibodies. The pSTAT1 band density was quantified with ImageJ and normalized to that of β-actin. Data were shown as mean ± S.D. n = 4. Welch’s *t*-test, **P < 0.01. (C) RNA samples were collected 24 hr after IFN-γ stimulation for qRT-PCR. The expression levels of the indicated genes were normalized to that of β-actin and shown as mean ± S.D. n = 5. One-way ANOVA, *P < 0.05, **P < 0.01.

Hepatocyte death induced by IFN-γ is dependent on STAT1 activation as described above. Therefore, we hypothesized that IL-11 pretreatment mitigated hepatocyte death by influencing STAT1 signaling. IFN-γ stimulation alone increased phosphorylated STAT1 (pSTAT1), which peaked 1 hr after stimulation and returned to an undetectable level by 24 hr (Fig 3A). At later time points, i.e., 8 hr and 24 hr, the total amount of STAT1 increased, instead of pSTAT1, due to transcriptional activation of STAT1 by itself [23,24]. It should be noted that IFN-γ stimulation also activated STAT3, the phosphorylation pattern of which traced the same time-dependent vicissitude as that of STAT1.

Next, IL-11 was used for pretreatment before IFN-γ stimulation. As a result, the intense STAT1 phosphorylation 1 hr post-IFN-γ stimulation was hampered by IL-11 pretreatment (Fig 3B). We also examined mRNA expression of STAT1-downstream molecules promoting cell death and revealed that IRF-1, caspase-1, bak, bax, and bim were upregulated by IFN-γ, which was significantly restrained by IL-11 pretreatment, except for bim (Fig 3C).

### Either IFN-γ or IL-11 stimulation upregulated SOCSs

**Fig 4:**
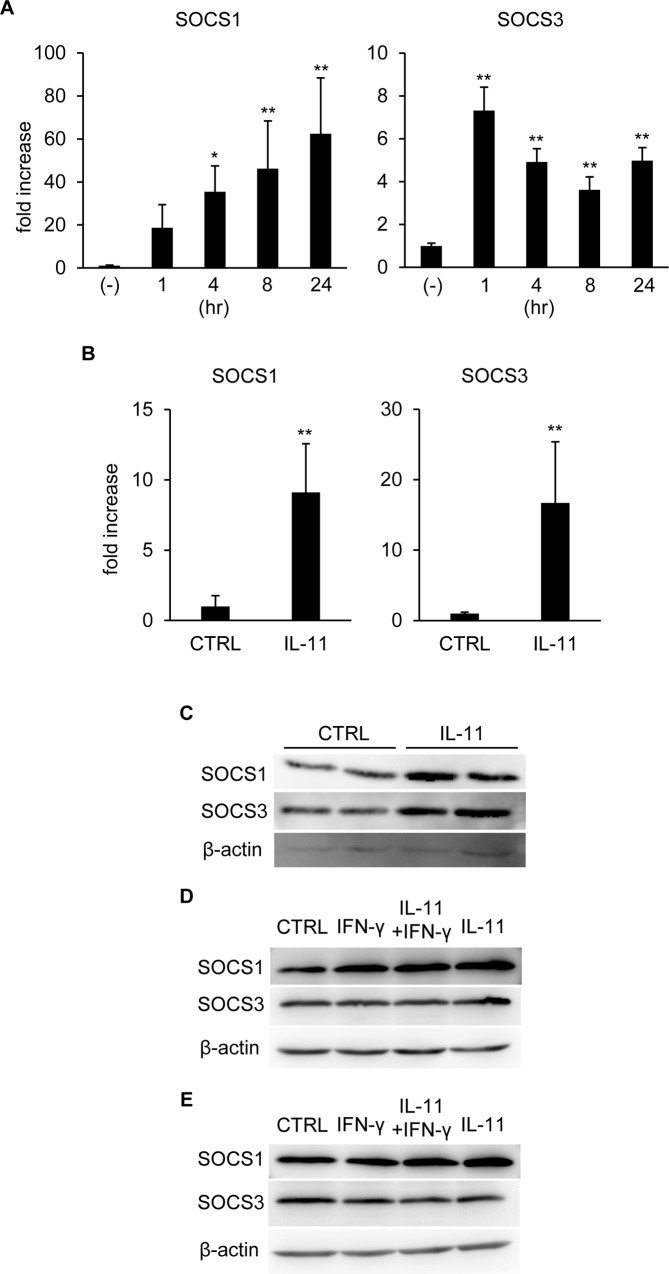
SOCSs expression was increased by either IFN-γ or IL-11. (A) qRT-PCR was performed for hepatocyte RNA samples collected at the indicated time points after IFN-γ stimulation. The expression levels of the indicated genes were normalized to that of β-actin and shown as mean ± S.D. n = 4. One-way ANOVA, *P < 0.05, **P < 0.01. (B) RNA samples collected from hepatocytes 16 hr after IL-11 stimulation were subjected to qRT-PCR. The expression levels of the indicated genes were normalized to that of β-actin and shown as mean ± S.D. n = 4. Welch’s *t*-test, **P < 0.01. (C) Hepatocytes were treated with IL-11 and protein samples collected 16 hr after IL-11 stimulation were subjected to immunoblotting with anti-β-actin, SOCS1, and SOCS3 antibodies. (D) Protein samples were collected 4 hr after IFN-γ stimulation with/without IL-11 pretreatment and subjected to immunoblotting with the indicated antibodies. (E) Protein samples were collected 24 hr after IFN-γ stimulation with/without IL-11 pretreatment and subjected to immunoblotting with the indicated antibodies.

In order to clarify the mechanism of prompt deactivation of STATs after IFN-γ stimulation, we examined expression of the suppressor of cytokine signaling (SOCS) family genes. mRNA of both SOCS1 and SOCS3, potent negative feedback regulators of IFN-γ/JAK/STAT signaling [25–27], was upregulated after IFN-γ stimulation (Fig 4A). Since IL-11 as well as IFN-γ, signals through JAK/STAT cascade, it is reasonable that IL-11 also upregulated mRNA of SOCSs (Fig 4B). We also examined protein expression of SOCSs after IFN-γ stimulation with/without IL-11 pretreatment. Consistent with mRNA expression, both SOCS1 and SOCS3 proteins were increased 16 hr after IL-11 treatment (Fig 4C). At 4 hr after IFN-γ stimulation, SOCS1 was induced in the IL-11 and/or IFN-γ-treated groups, while SOCS3 protein induction was hardly observed (Fig 4D). In the IL-11-treated groups, upregulation of SOCS1 was sustained until 24 hr after IFN-γ stimulation. On the other hand, SOCS1 protein induction was terminated at this time point when only IFN-γ was applied, although mRNA expression remained at a high level (Fig 4A and 4E). Taken together, SOCSs, although mRNA and protein expression patterns may not completely correspond, are upregulated upon activation of STATs, mediating negative feedback regulation of IFN-γ signaling, but not IL-11.

### IFN-γ pretreatment failed to affect STAT3 activation by IL-11

**Fig 5:**
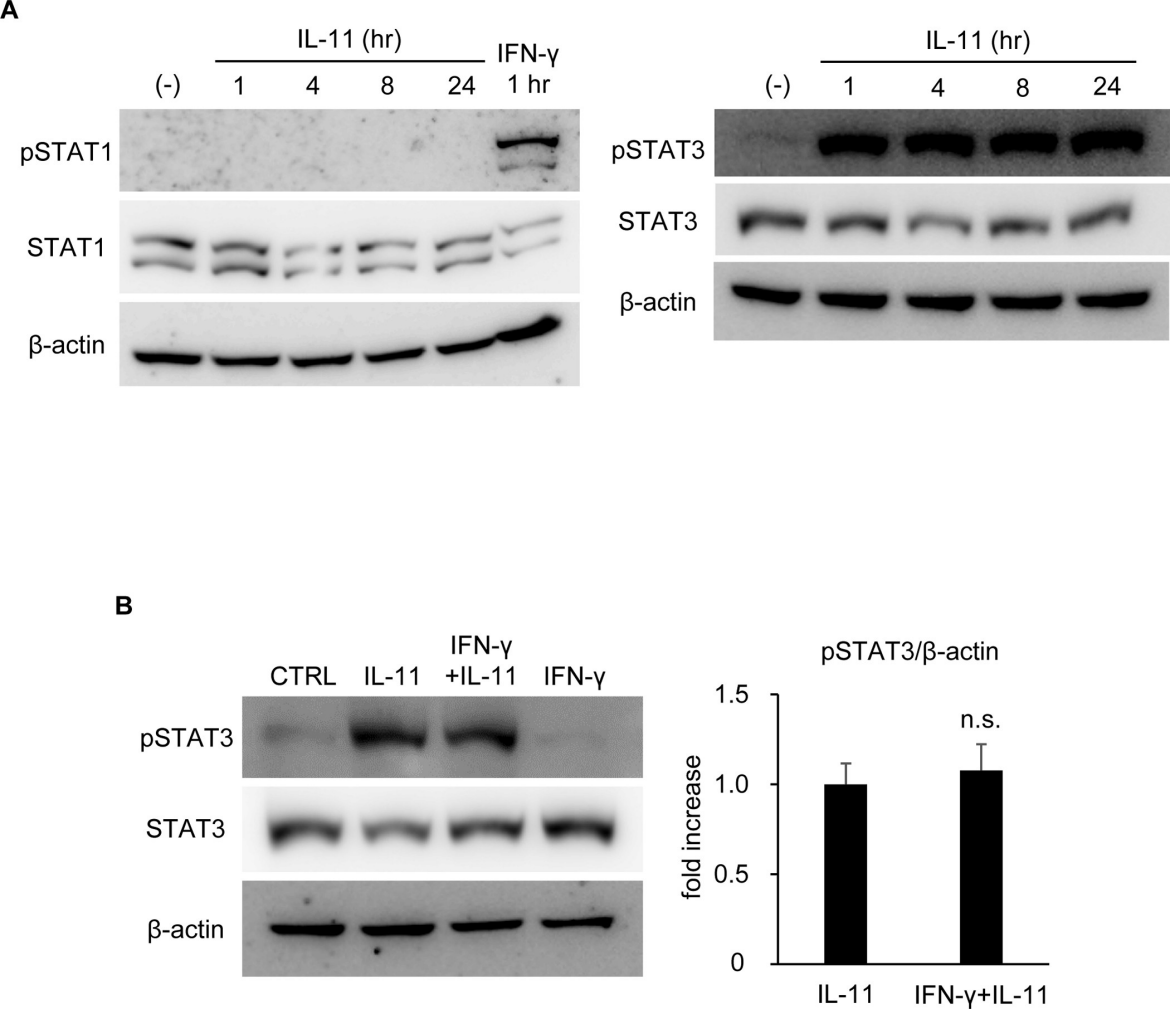
IFN-γ pretreatment showed no mitigating effect on IL-11-induced STAT3 activation. (A) Hepatocytes were stimulated by IL-11. Protein samples were collected at the indicated time points after stimulation for immunoblotting with anti-β-actin, STAT1, pSTAT1, STAT3, and pSTAT3 antibodies. A protein sample collected 1 hr after IFN-γ stimulation was applied as a positive control of pSTAT1. (B) Hepatocytes were pretreated with IL-11 for 16 hr, followed by IFN-γ stimulation. Protein samples collected 1 hr after IFN-γ stimulation were subjected to immunoblotting with anti-β-actin, STAT3, and pSTAT3 antibodies. The pSTAT3 band density was quantified with ImageJ and normalized to that of β-actin. Data were shown as mean ± S.D. n = 4. Welch’s *t*-test, n.s.: not significant.

In order to fully clarify the signal cross-regulation between IFN-γ and IL-11, we further tested the effect of IFN-γ pretreatment on IL-11-induced STAT3 activation. When hepatocytes were stimulated with IL-11 alone, STAT3, but not STAT1, was strongly activated (Fig 5A). It is of note that the activation of STAT3 was observed immediately after stimulation and prolonged at least until 24 hr without apparent decline, which is a remarkable difference from IFN-γ signaling. Even when IFN-γ was used for pretreatment, IL-11-induced STAT3 activation was unaffected (Fig 5B). Considering that both IFN-γ and IL-11 upregulated SOCSs but only IFN-γ signaling was transient and was suppressed by pretreatment of the other, it is conceivable that negative feedback regulation by SOCSs exhibits a preference for IFN-γ signaling to IL-11 signaling in hepatocytes.

### IL-11 conferred oxidative stress resistance to hepatocytes

**Fig 6:**
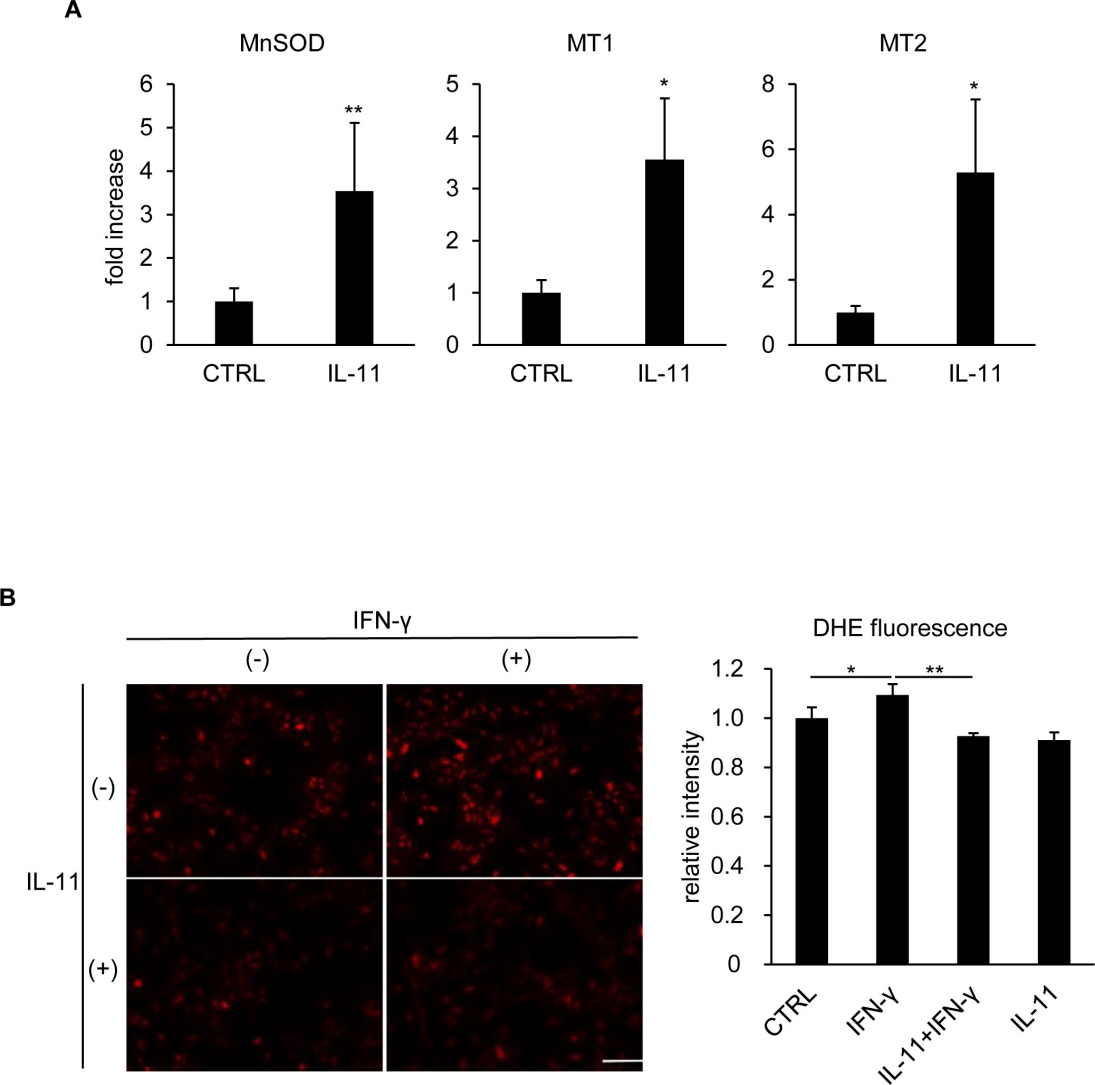
Oxidative stress was reduced by IL-11 pretreatment. (A) Hepatocytes were stimulated by IL-11. RNA samples collected 16 hr after stimulation were subjected to qRT-PCR. The expression level of the indicated genes was normalized to that of β-actin and shown as mean ± S.D. n = 4. Welch’s *t*-test, *P < 0.05, **P < 0.01. (B) Hepatocytes were pretreated with IL-11 for 16 hr, followed by IFN-γ stimulation. DHE assay was performed 8 hr after IFN-γ stimulation. Bar: 100 μm. DHE fluorescence was quantified with ImageJ and shown as relative intensity. Fluorescence of approximately 16000 cells in total from four independent pictures was quantified for each group. One-way ANOVA, *P < 0.05, **P < 0.01.

We have previously reported that IFN-γ stimulation induces oxidative stress, which is also required for hepatocyte death [28], while IL-11/STAT3 signaling is known to promote ROS resistance [17]. As another aspect of hepatocyte protection by IL-11, we finally investigated whether hepatocytes acquired ROS resistance after IL-11 treatment. mRNA expression of IL-11/STAT3-downstream ROS scavengers, MnSOD, MT1, and MT2 [15,16], were found to be increased by IL-11 (Fig 6A). Intracellular ROS levels were analyzed by DHE, the oxidized form of which emits red fluorescence. IFN-γ stimulation alone significantly increased ROS in hepatocytes, which was completely blocked by IL-11 pretreatment (Fig 6B). Taken together, IL-11 pretreatment protected hepatocytes from IFN-γ-induced death through two distinct mechanisms: downregulating STAT1 signaling and conferring ROS resistance.

## Discussion

Although IL-11 has been known to protect the liver in the contexts of acetaminophen-induced and ischemia/reperfusion injury [12–14,17], its role in cytokine-induced hepatocyte damage remained unrevealed. In this study, we addressed the effects of IL-11 on hepatocyte death induced by IFN-γ. As a result, IL-11 pretreatment significantly attenuated IFN-γ-induced hepatocyte death by suppressing STAT1 signaling, while IFN-γ pretreatment showed a negligible effect on IL-11-induced STAT3 activation. Additionally, IL-11 enhanced the ROS scavenging capacity of hepatocytes, which could further support its protective function.

IFN-γ exhibits its hepatotoxic effects from relatively later time points, i.e., 24 hr after stimulation, indicating that secondary or even latter stage effectors, including IRF-1, are essential for promoting cell death [5,18,29]. As such, it is possible that the increase in unphosphorylated STAT1 observed from 8 hr after IFN-γ stimulation (Fig 3A) also played a role in damaging hepatocytes. In human fibroblasts or mammary epithelial cells, for example, unphosphorylated STAT1 accumulating in response to IFN-β or IFN-γ is important in maintaining expression of a subset of IFN-responsive genes [30]. Actually, we found that IRF-1, which was upregulated from as early as 1 hr after IFN-γ stimulation, remained at a high level at least until 48 hr (S2 Fig), though pSTAT1 had returned to an inactive state much earlier. Thus, IFN-γ-induced hepatocyte death may still harbor undisclosed mechanistic aspects including the relevance of unphosphorylated STAT1. However, IL-11 pretreatment is expected to interfere with these cytotoxic machineries comprehensively, since it hampered STAT1 phosphorylation, a major primary phase of IFN-γ-mediated cytotoxicity.

IL-11 is known to exhibit its protective function through not only STAT3 but also other signaling cascades, such as Akt and ERK. Actually, we investigated the activation status of known IL-11-downstream molecules [31,32] and found that Akt and ERK, but not p38 were phosphorylated upon IL-11 treatment (S3 Fig). Although it is possible that these molecules as well as STAT3 are coordinately mediating hepatocyte protection, we focused particularly on STAT signaling, since we believe that one of the most interesting findings in this study is the selective negative feedback regulation of STAT signaling. There are several possible interpretations for this selectivity. IFN-γ receptors and IL-11 receptors are associated with different subtypes of JAK from each other: JAK1 and JAK2 for IFN-γ and JAK1 alone for IL-11 [33]. Therefore, a hypothesis that SOCSs preferentially target JAK2 relative to JAK1 seems to be an attractive explanation for the selective IFN-γ signal inhibition. However, this idea is partially falsified by previous reports demonstrating that SOCS1 inhibits both JAK1 and JAK2 [34,35]. Furthermore, STAT3 phosphorylation by IL-6, utilizing only JAK1 under the receptor like IL-11, is suppressed by IFN-γ pretreatment in human endothelial cells [36]. Considering that IL-11 signaling was unaltered by IFN-γ pretreatment in our experiments, the JAK difference by itself cannot fully explain the selective negative feedback regulation of cytokine signaling, although species or cell type variation may be another key factor that determines which signaling is to be suppressed.

Since SOCSs bind to receptor-JAK complex rather than a JAK monomer in many situations [37–39], another possible model of the selective negative feedback regulation of IFN-γ signaling is that SOCSs have different affinities to receptor-JAK complex depending on the receptor type. Croker et al., using SOCS-deficient mouse hepatocytes, have studied specificity of SOCS1 and SOCS3 in inhibiting IFN-γ and IL-6 signaling and shown that SOCS1 is specific to IFN-γ, while SOCS3 is to IL-6 [40]. In our experiments, IL-11 induced sustained STAT3 activation despite upregulation of SOCSs, and IFN-γ signaling was downregulated by IL-11 pretreatment. Magrangeas et al. have featured signal crosstalk between IL-3 and IL-11 in Ba/F3 cells engineered to express IL-11 receptor [41]. They reported that IL-3 pretreatment inhibited STAT3 activation by subsequent IL-11 stimulation through STAT5/SOCS3 cascade, whereas IL-11 pretreatment did not affect IL-3-induced STAT5 activation. Taken together, IL-11 receptor-JAK complex is not totally but relatively insensitive to SOCSs compared to IL-6 or IFN-γ receptor-JAK complex.

From the viewpoint of biological significance, it may be reasonable that IL-11 signaling overwhelms cytotoxic signal pathways. In the liver, IL-11 is known to be expressed from hepatocytes in response to oxidative stress [17,42], which could define the dominance of IL-11 over cytotoxic stimuli as a brake of hepatic damage to maintain tissue homeostasis. Future investigation into the selective negative feedback regulation of cytokine signals will lead to further understanding of molecular systems, which balance hepatocyte survival and death.

To conclude, we revealed for the first time the protective and dominant effect of IL-11 on hepatocytes challenged by IFN-γ, shedding new light on hepatocyte molecular biology.

## Supporting information

S1 Fig Caspase 3 activation after IFN-γ stimulation was attenuated by IL-11 pretreatment. Hepatocytes were pretreated with IL-11, followed by IFN-γ stimulation 16 hr after IL-11 pretreatment. Protein samples collected 24 hr after IFN-γ stimulation were subjected to immunoblotting with anti-Caspase 3 (9662, CST) antibody.
(DOCX)

S2 Fig IRF-1 expression remained at a high level until 48 hr after IFN-γ stimulation. qRT-PCR was performed for hepatocyte RNA samples collected at the indicated time points after IFN-γ stimulation. The expression levels of the indicated genes were normalized to that of β-actin and shown as mean ± S.D. n = 4. One-way ANOVA, *P < 0.05, **P < 0.01.
(DOCX)

S3 Fig Akt and ERK were activated after IL-11 treatment. Hepatocytes were treated with IL-11 and protein samples collected 1 hr after IL-11 stimulation were subjected to immunoblotting with indicated anti-pAkt (9271, CST), anti-Akt (9272, CST), anti-p-p38 (9211, CST), anti-p38 (8690, CST), anti-pERK (4370, CST), anti-ERK (9102, CST) and anti-β-actin antibodies.
(DOCX)

S4 Fig Uncropped immunoblot images of main figures. (DOCX)

S1 Table Mean values and standard deviations. (DOCX)
