# Supplementary material for: IL-11 prevents IFN-γ-induced hepatocyte death through selective downregulation of IFN-γ/STAT1 signaling and ROS scavenging
Source: PLoS One. 2019 Feb 19;14(2):e0211123. doi: 10.1371/journal.pone.0211123 (PMC6380568; doi:10.1371/journal.pone.0211123)
Supplement: S1 Fig — Hepatocytes were pretreated with IL-11, followed by IFN-γ stimulation 16 hr after IL-11 pretreatment. Protein samples collected 24 hr after IFN-γ stimulation were subjected to immunoblotting with anti-Caspase 3 (9662, CST) antibody. (DOCX) [file pone.0211123.s001.docx]

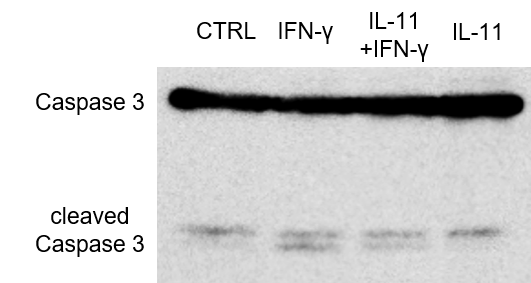


**S1 Fig**

**Caspase 3 activation after IFN-γ stimulation was attenuated by IL-11 pretreatment.**

Hepatocytes were pretreated with IL-11, followed by IFN-γ stimulation 16 hr after IL-11 pretreatment. Protein samples collected 24 hr after IFN-γ stimulation were subjected to immunoblotting with anti-Caspase 3 (9662, CST) antibody.
