# Supplementary material for: IL-11 prevents IFN-γ-induced hepatocyte death through selective downregulation of IFN-γ/STAT1 signaling and ROS scavenging
Source: PLoS One. 2019 Feb 19;14(2):e0211123. doi: 10.1371/journal.pone.0211123 (PMC6380568; doi:10.1371/journal.pone.0211123)
Supplement: S2 Fig — qRT-PCR was performed for hepatocyte RNA samples collected at the indicated time points after IFN-γ stimulation. The expression levels of the indicated genes were normalized to that of β-actin and shown as mean ± S.D. n = 4. One-way ANOVA, *P < 0.05, **P < 0.01. (DOCX) [file pone.0211123.s002.docx]

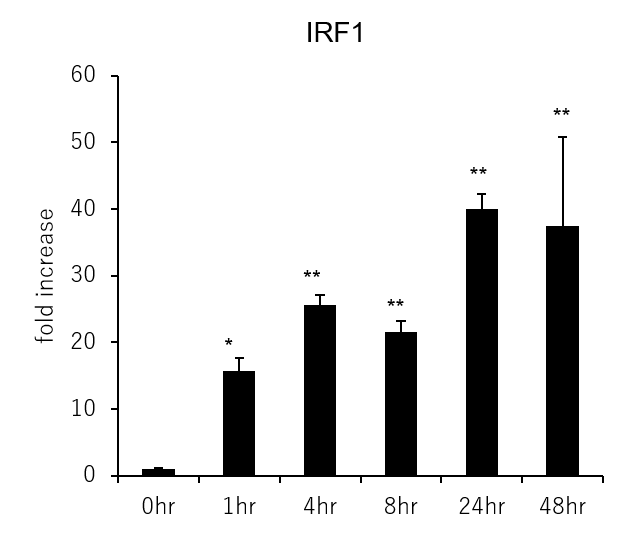


**S2 Fig**

**IRF-1 expression remained at a high level until 48 hr after IFN-γ stimulation.**

qRT-PCR was performed for hepatocyte RNA samples collected at the indicated time points after IFN-γ stimulation. The expression levels of the indicated genes were normalized to that of β-actin and shown as mean ± S.D. n = 4. One-way ANOVA, ^*^*P* < 0.05, ^**^*P* < 0.01.
