# Supplementary material for: IL-11 prevents IFN-γ-induced hepatocyte death through selective downregulation of IFN-γ/STAT1 signaling and ROS scavenging
Source: PLoS One. 2019 Feb 19;14(2):e0211123. doi: 10.1371/journal.pone.0211123 (PMC6380568; doi:10.1371/journal.pone.0211123)
Supplement: S3 Fig — Hepatocytes were treated with IL-11 and protein samples collected 1 hr after IL-11 stimulation were subjected to immunoblotting with indicated anti-pAkt (9271, CST), anti-Akt (9272, CST), anti-p-p38 (9211, CST), anti-p38 (8690, CST), anti-pERK (4370, CST), anti-ERK (9102, CST) and anti-β-actin antibodies. (DOCX) [file pone.0211123.s003.docx]

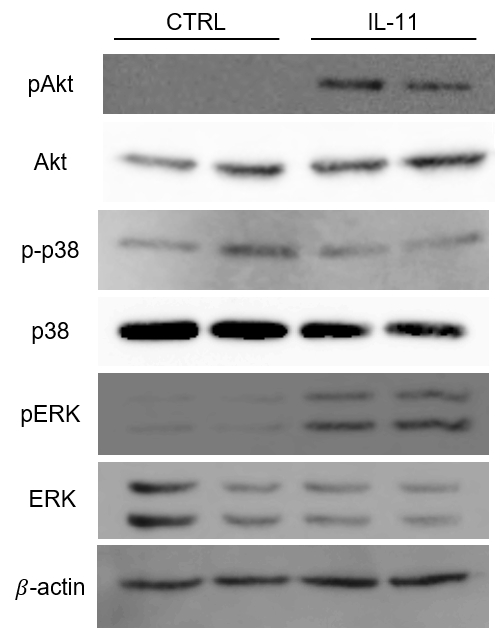


**S3 Fig**

**Akt and ERK were activated after IL-11 treatment.**

Hepatocytes were treated with IL-11 and protein samples collected 1 hr after IL-11 stimulation were subjected to immunoblotting with indicated anti-pAkt (9271, CST), anti-Akt (9272, CST), anti-p-p38 (9211, CST), anti-p38 (8690, CST), anti-pERK (4370, CST), anti-ERK (9102, CST) and anti-β-actin antibodies.
