# Supplementary figures and images for: IL-11 prevents IFN-γ-induced hepatocyte death through selective downregulation of IFN-γ/STAT1 signaling and ROS scavenging
Source: PLoS One. 2019 Feb 19;14(2):e0211123. doi: 10.1371/journal.pone.0211123 (PMC6380568; doi:10.1371/journal.pone.0211123)

**S4 Fig**

**Uncropped immunoblot images of main figures.**


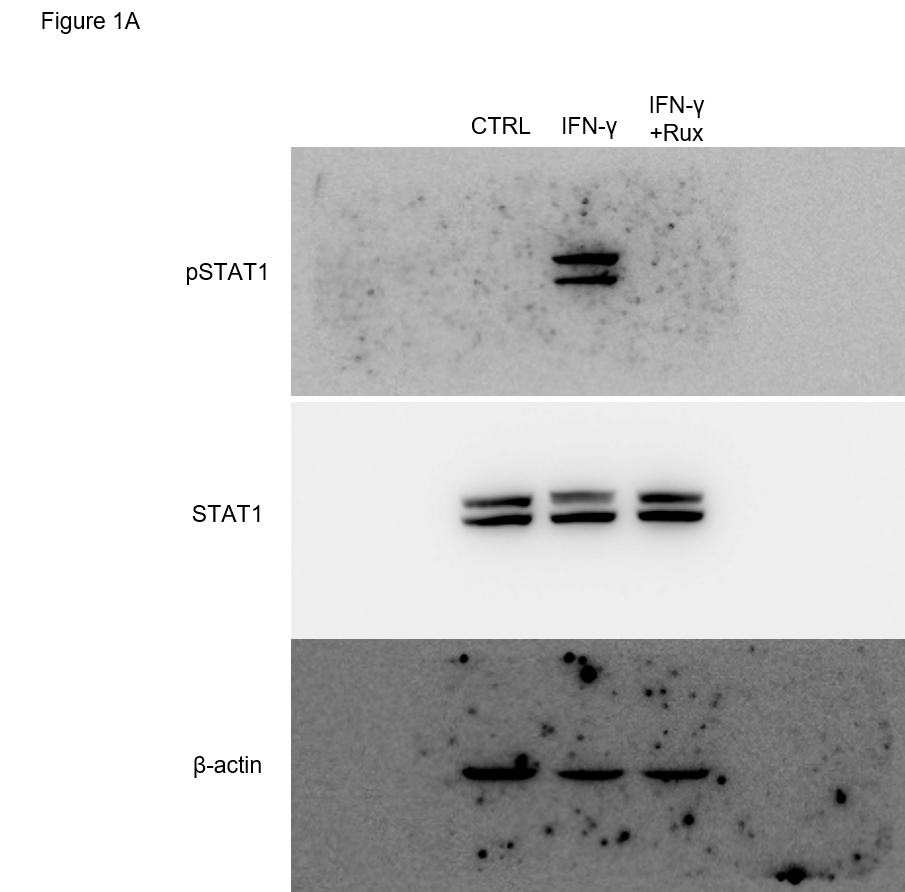


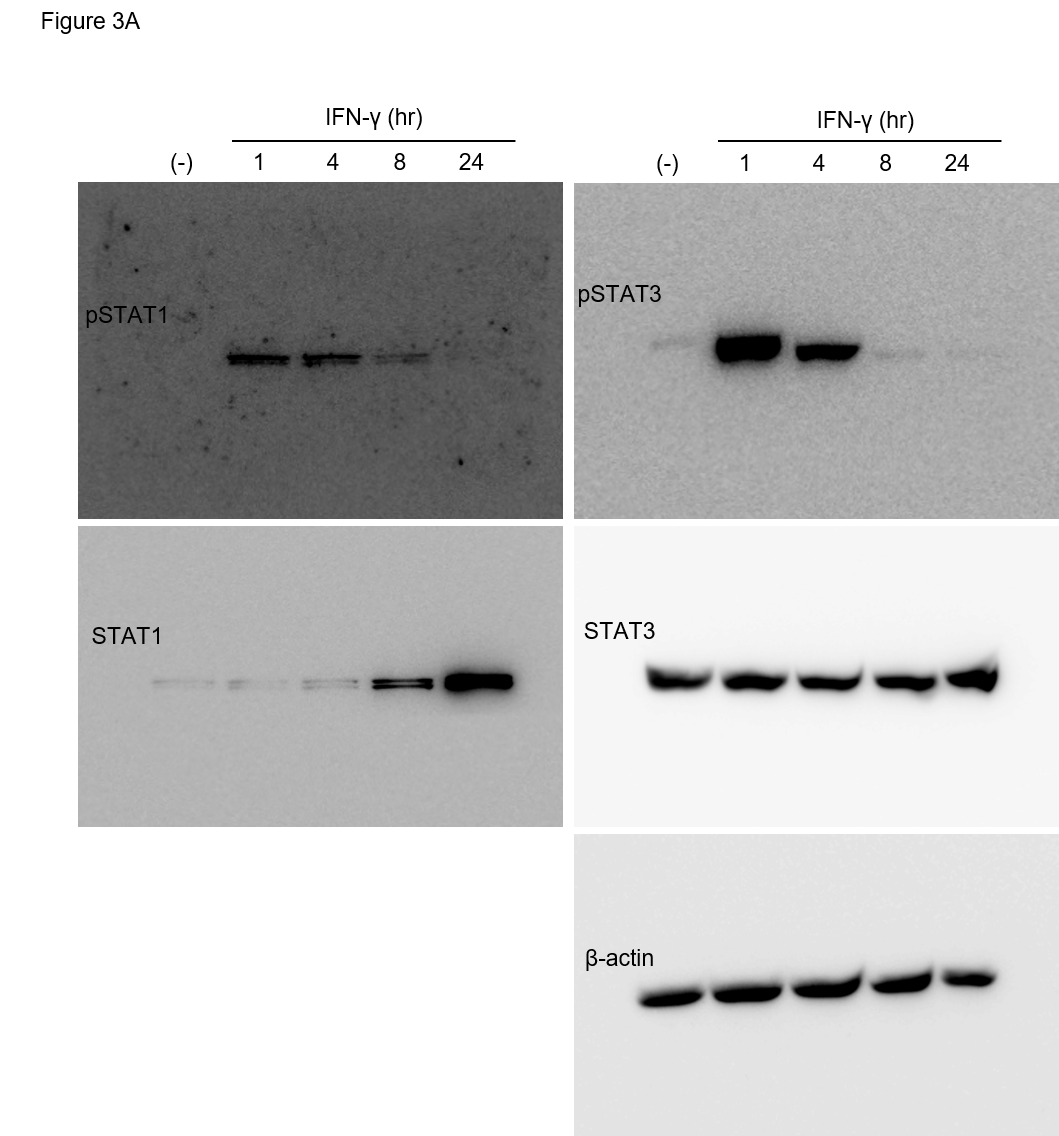


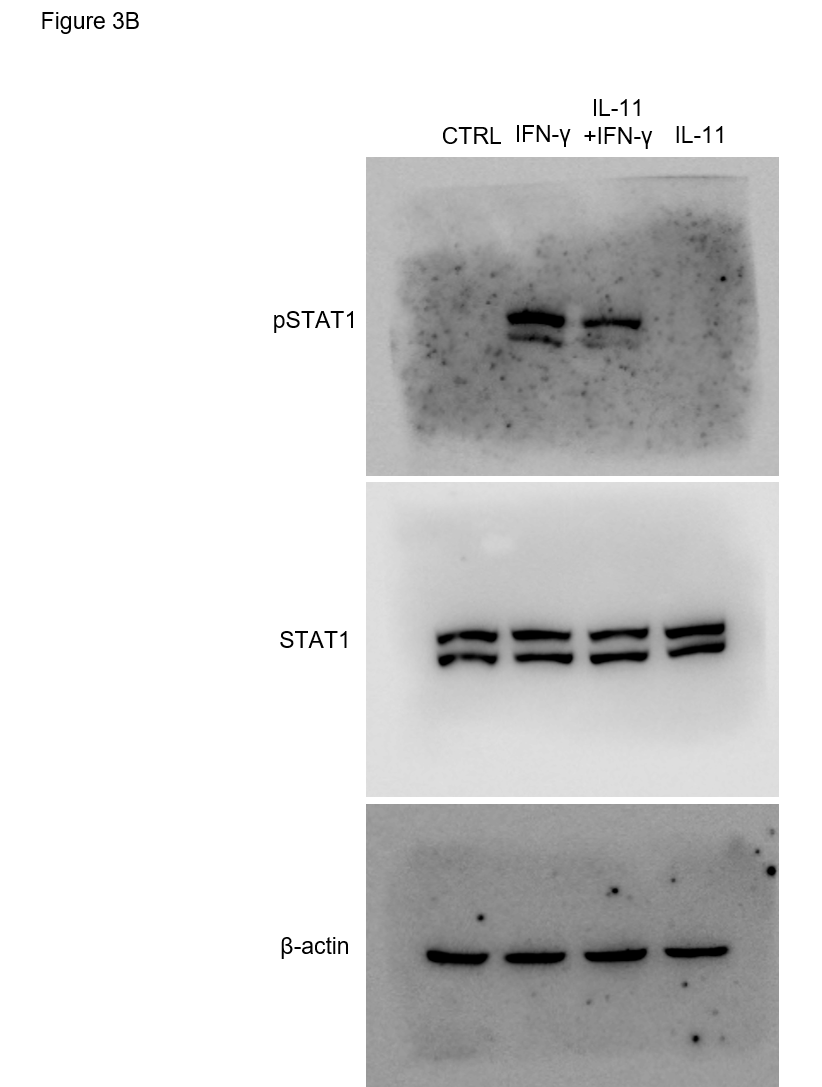


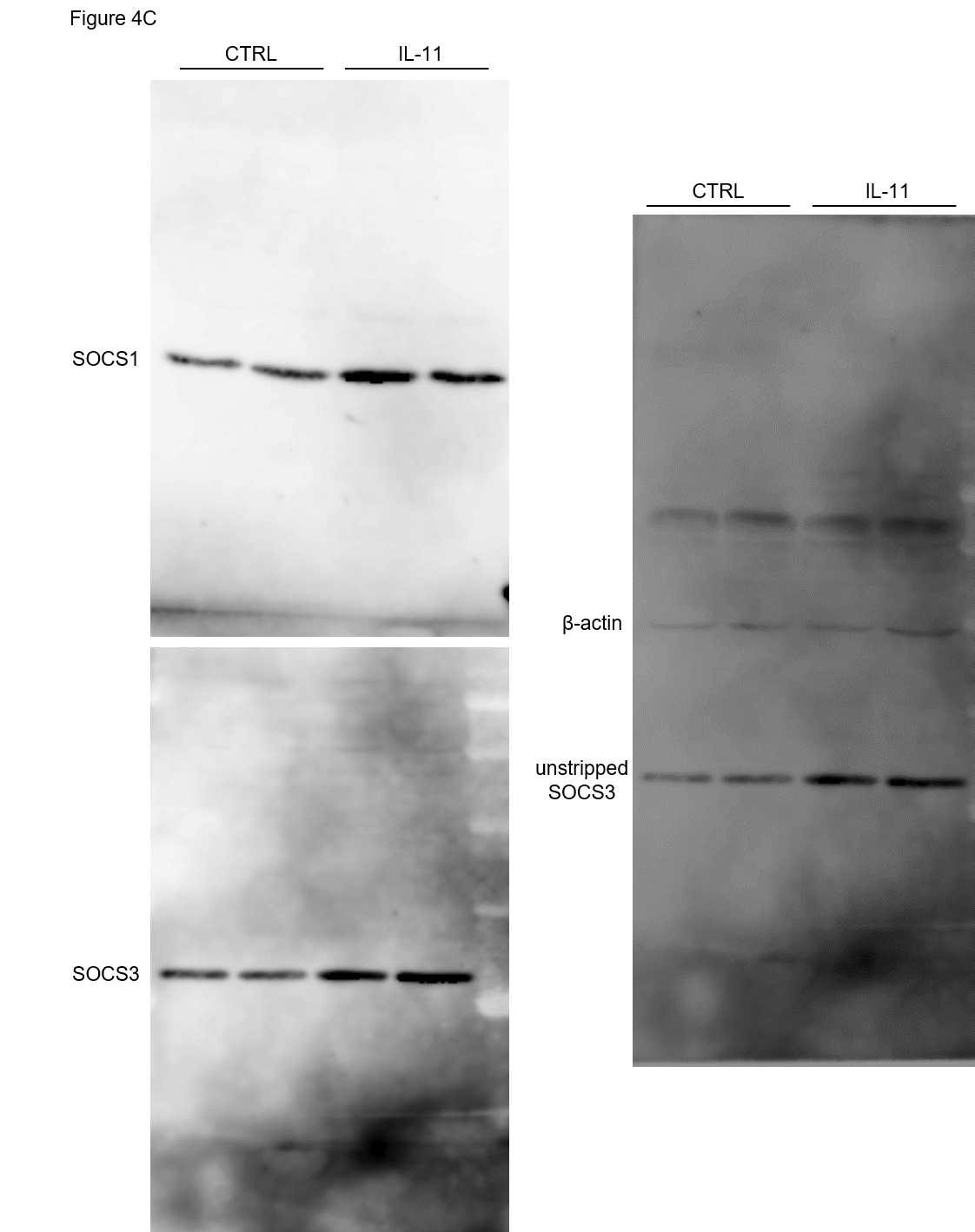


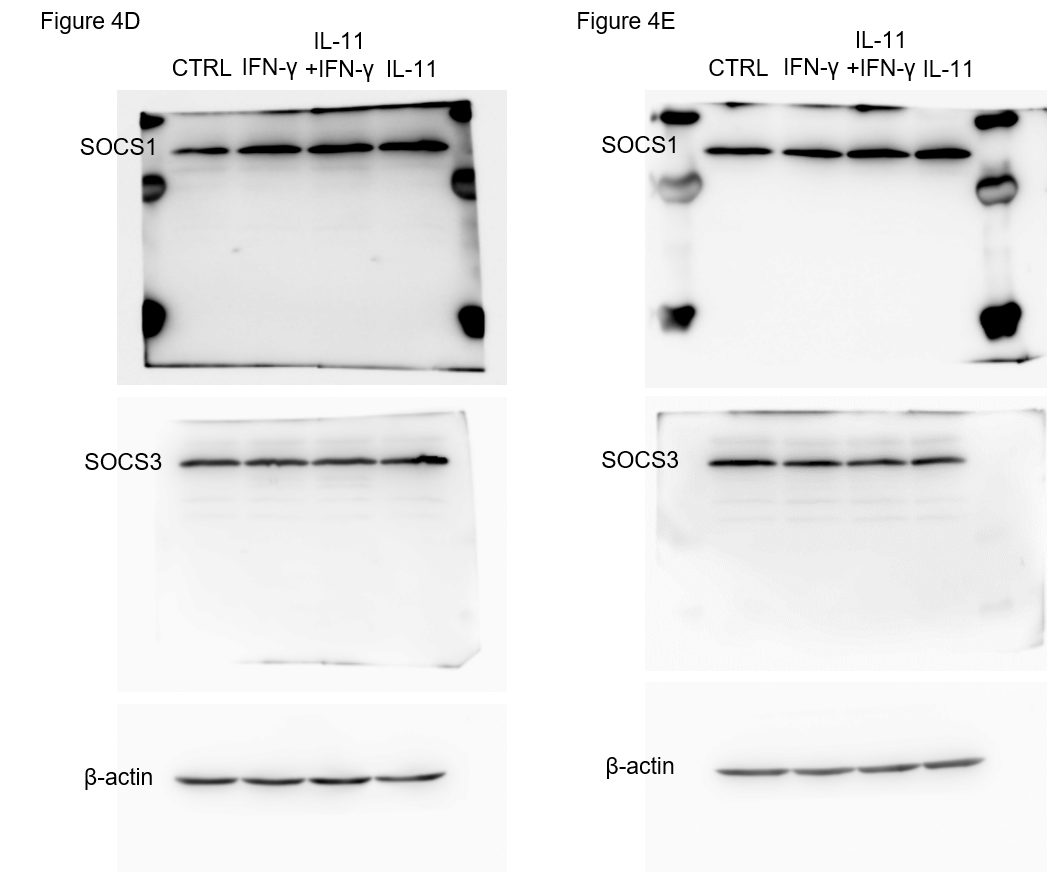


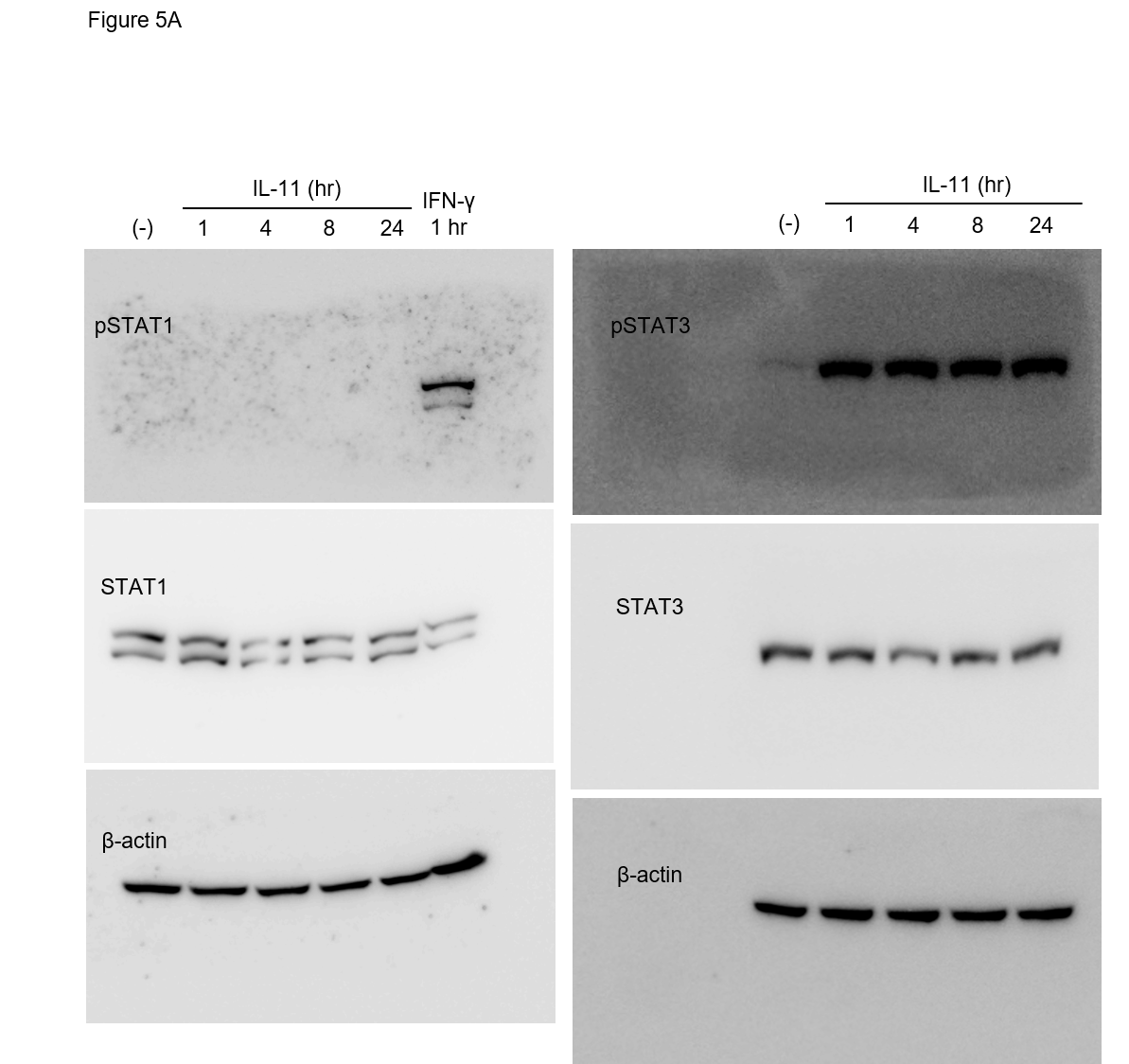


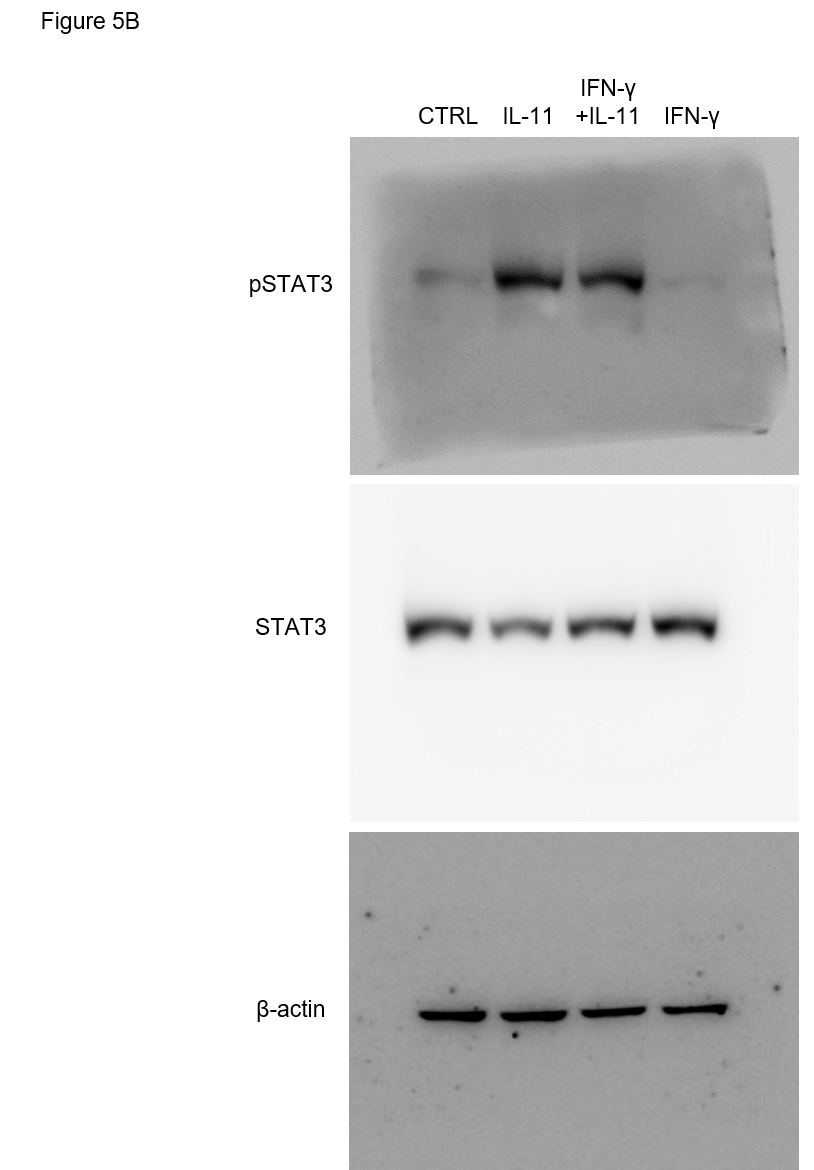

Supplement: S4 Fig — (DOCX) [file pone.0211123.s004.docx]
