# Supplementary material for: IL-11 prevents IFN-γ-induced hepatocyte death through selective downregulation of IFN-γ/STAT1 signaling and ROS scavenging
Source: PLoS One. 2019 Feb 19;14(2):e0211123. doi: 10.1371/journal.pone.0211123 (PMC6380568; doi:10.1371/journal.pone.0211123)
Supplement: S1 Table — (DOCX) [file pone.0211123.s005.docx]

**S1 Table**

**Mean values and standard deviations.**

All the data used for calculating mean and S.D. values are presented.
